# Co-Operation Centres, Prescribing and Fees

**Published:** 1914-06-13

**Authors:** 


### THE LONDON PANEL COMMITTEE.

### Co-operation Centres, Prescribing and Fees.

The London Panel Committee on June 9 again had before it the question of the best ways in which the money provided in the Budget in aid of public health and health insurance services could be expended, from the point of view of the medical profession and of insured persons. The Panel Service Sub-Committee advised that, as the character and scope of the proposed centres for the co-operation of practitioners had not been defined the Panel Committee should place its opinions before the Insurance Commissioners. The Sub-Committee felt that in many areas in London centres to which practitioners might take difficult cases for discussion with other practitioners would not be widely used. The conditions of medical practice differed so much in various parts of London that it was difficult to formulate a scheme which would be satisfactory everywhere, but as an experiment the money available might well be utilised for the establishment of centres where a number of practitioners would be able to see their industrial patients. This would apply especially in areas where it was difficult for doctors individually to secure satisfactory surgery and waiting-room accommodation, and the use of such centres in common would make it possible to engage the services of a clerk, an *x*-ray operator, consultant, and medical referees. It might even be possible to secure that one or more trained nurses should be attached to the centre, as well as a chemist who was on the list of persons supplying drugs and appliances. The Sub-Committee made recommendations on the lines of these suggestions, but as the report was not reached until the end of a long sitting consideration had to be adjourned because there was not a *quorum* present.

A discussion arose in which several members deprecated the idea that the Panel Committee should sit in judgment on certain practitioners against whom excessive or extravagant prescribing was alleged; for example, the ordering of cylinders of oxygen, and musk at 10s. a dose. It was pointed out, however, that the Panel Committee had a statutory duty to inquire into such allegations, and a member remarked that it was no more than occurred in every hospital where the prescriptions ordered by consultants were noted and any which were deemed excessive were criticised by other members of the medical staff.

The Committee approved a scheme for the allocation of persons who had been refused by the doctors whom they selected, and for the allotment of the capitation fees in respect of such persons. In the debate some heat was aroused when Dr.

D. F. Keenan, Hackney, denounced the arrangement made by the Insurance Committee that no further capitation fees should be credited to a doctor in respect of insured persons on his list beyond 2,000. Dr. Keenan described this as a swindle and right-down thieving, and he condemned the medical representatives on the Insurance Committee for having assented to it.

Dr. B. A. Richmond replied that the medical representatives fought for the freedom of the doctor in this matter, but they were outvoted, and the figure 2,000 was accepted in preference to 1,500.

